# The Adhesive Perinephric Fat Score is Correlated with Outcomes of Retroperitoneal Laparoscopic Adrenalectomy for Benign Diseases

**DOI:** 10.1007/s00268-022-06671-1

**Published:** 2022-08-01

**Authors:** Wei Chen, Qixiang Fang, Shangshu Ding, Xiaonan Wu, Pan Zhang, Jing Cao, Dapeng Wu

**Affiliations:** 1 Department of Urology, The First Affiliated Hospital of Xi’an Jiaotong University, #277 West Yanta Road, Xi’an, 710061 Shaanxi Province People’s Republic of China; 2 Department of Urology, Shaanxi Provincial People’s Hospital, Xi’an, 710068 People’s Republic of China; 3 Department of Infectious Diseases, The First Affiliated Hospital of Xi’an Jiaotong University, Xi’an, 710061 People’s Republic of China

## Abstract

**Background:**

Retroperitoneal laparoscopic adrenalectomy (RLA) possessing unique superiority with minimal abdominal interference is complicated by the status of periadrenal fat, including its quantity and texture. We hypothesized that an adherent perinephric fat predictor, the Mayo Adhesive Probability score (Mayo score), is associated with the perioperative outcomes of RLA.

**Methods:**

This retrospective study included consecutive patients who underwent RLA for the diagnosis of benign adrenal tumors at our institution between 2017 and 2020. Medical records were reviewed to evaluate the association between Mayo scores obtained from preoperative computed tomography imaging and surgical outcomes as well as complications. Factors independently related to perioperative results were analyzed using multivariable regression models.

**Results:**

In total, 186 RLA were included. According to their Mayo scores, the patients were divided as follows: 0 (*n* = 51, 27.4%), 1 (*n* = 34, 18.3%), 2 (*n* = 45, 24.2%), 3 (*n* = 29, 15.6%), 4 (*n* = 16, 8.6%) and 5 (*n* = 11, 5.9%). Longer operative time (92.0 ± 25.0 vs. 114.7 ± 30.6 vs. 137.4 ± 27.1 min, *P* < 0.001), higher estimated blood loss (42.2 ± 28.1 vs. 70.5 ± 44.9 vs. 132.6 ± 63.4 mL, *P* < 0.001) and greater decline of hemoglobin (0.7 ± 0.4 vs. 1.0 ± 0.4 vs. 1.3 ± 0.6 g/dL, *P* < 0.001) were significantly associated with elevated Mayo score risks. No difference in complication rates was found. The score was identified as a unique, independent risk factor for perioperative outcomes on multivariable analysis.

**Conclusions:**

The Mayo score is a vital outcome predictor of RLA. It may be utilized in the preoperative planning for patients undergoing RLA.

## Introduction

Since its inception in 1992, laparoscopic adrenalectomy has promptly transformed the surgical status of nonmalignant adrenal tumors because of its remarkable advantages in terms of minimal invasiveness compared with the open approach and has become the gold standard treatment [1, 2]. With the development of instruments and techniques, RLA has gradually shown its unique superiority by just mobilizing ipsilateral kidney rather than other adjacent organs, thereby minimizing interference to them [3].

In the retroperitoneal approach, exposure of the adrenal gland involves three surrounding avascular planes, including the dissection of periadrenal fat and mobilization of the upper pole of kidney, even to the hilum [4]. Due to limited space, the characteristics of periadrenal fat dramatically affect the difficulty of exposure. Given the rapidly increasing global prevalence of overweight, obesity has always been identified as a risk factor for perioperative complications. Several reports have documented that obesity or increased body mass index (BMI) is a predictor of perioperative outcomes of RLA [5, 6]. However, some studies have reported contradictory results [7, 8]. Notwithstanding the amount of periadrenal fat concerned, there has been few reports regarding its texture.

The presence of adherent perinephric fat, which is difficult to dissect from the renal capsule, causes enormous time consumption and poses great challenges to kidney procedures, such as partial nephrectomy [9]. While the underlying pathogenesis is unclear, it has been suggested that inflammation and cardiovascular risk factors may account for adherent perinephric fat [10]. As an endocrine and immune organ, the role of adipose tissue in the development of chronic systemic inflammation has been emphasized in obesity related to insulin resistance and lipid dysregulation [11, 12]. Particularly in metabolic syndrome, an activated cascade of chemokines leads to the infiltration of macrophages into visceral fat and mediates the development of fibrosis and adhesion of perinephric fat [13].

The Mayo Adhesive Probability score (Mayo score) is an image-based scoring system initially used to preoperatively evaluate the possibility of encountering problematic adherent fat during robot-assisted partial nephrectomy. This ready-to-use risk score includes only two radiological factors, posterior perinephric fat thickness and perinephric fat stranding type [14]. The profile of perinephric fat, which can be reflected by Mayo score, is involved in the procedure of RLA.

The aim of our study was to determine whether Mayo score is correlated with perioperative outcomes of RLA performed for benign adrenal diseases. Accurate evaluation of perinephric fat status can practically guide education, consultation, surgical planning and, potentially, result estimation.

## Materials and methods

This study was approved by the Institutional Review Board of the First Affiliated Hospital of Xi’an Jiaotong University. The data of consecutive patients who received RLA for adrenal tumors between August 2017 and March 2020 at our institution were retrospectively reviewed. Patients with transperitoneal adrenalectomy, maximal tumor diameter > 5 cm, adrenal malignancy, pheochromocytomas, paragangliomas, partial adrenalectomy or prior ipsilateral retroperitoneal surgery were excluded.

The collected data included demographics (age, sex, BMI, American Society of Anesthesiologists (ASA) score and Charlson Comorbidity Index (CCI)), tumor characteristics (tumor size, laterality, presenting symptoms and pathological results), and perioperative outcomes. Operative time, estimated blood loss (EBL), decline of hemoglobin (DHb, defined as the change in hemoglobin values in routine blood tests between postoperative day 1 and preoperative day) were recorded as the perioperative outcomes. Complications classified by the Clavien–Dindo system and drainage tube removal time were also collected. Follow-up was performed 1 month after surgery and every 6 months for the next 2 years.

### Operative technique

All procedures were performed by two experienced surgeons in our department (D.W. and W.C.). Briefly, patients were placed in the lateral decubitus position. The procedure was performed using a 3-trocar technique. The three avascular planes were dissected successively under guidance, as previously described [4]. The first dissection plane between perinephric fat and anterior renal fascia was located on the superomedial side of upper renal pole. The plane between perinephric fat and posterior renal fascia was then separated. The third plane was adjacent to the surface of upper pole parenchyma. If thick perinephric fat was encountered, it was sometimes necessary to remove part of it to achieve better space and vision. The key step was to clip and transect the central adrenal vein. The isolated adrenal gland was retrieved through the postaxillary port in the entrapment sack. Complete hemostasis was confirmed under lowered air pressure (8 mmHg).

### Evaluation of Mayo score

By reviewing cross-sectional images electronically, Mayo score was determined using two variables, including thickness of posterior perinephric fat and perinephric stranding type on the ipsilateral side. The thickness was measured as a direct line posteriorly from renal capsule to the inner side of abdominal wall. Perinephric stranding, defined as the stripe of soft tissue attenuation in the perinephric area, was graded as no stranding, type 1 (thin mild stranding), or type 2 (diffuse, thick-banded severe stranding). The individual scores for the two variables were then summed to obtain the Mayo score (range 0–5).

### Statistical analysis

To compare the outcomes among the risk groups, analysis of variance (ANOVA) was used to analyze continuous variables, and the results were shown as means with standard deviations (SDs). Pearson’s chi-squared test was used for categorical variables, and the results were presented as numbers. Clinical factors, including age, sex, BMI, lesion size, manifestation and CCI, were evaluated to analyze their correlation with Mayo score using logistic regression models. The association between clinically relevant patient characteristics, including Mayo score, and perioperative outcomes was assessed using univariable and multivariable linear regression models. For the complication analysis, a logistic regression model was used. The selection of the variables included in the models incorporated clinical experience, previous reports and univariable analysis results. All variables included in the multivariable analysis had *P* values less than 0.20 in the univariable analysis. Statistical analyses were performed using SPSS version 20.0 (SPSS Inc., Chicago, IL, USA). All reported *P* values were two-sided, with *P* < 0.05 indicating statistical significance.

## Results

**Fig. 1:**
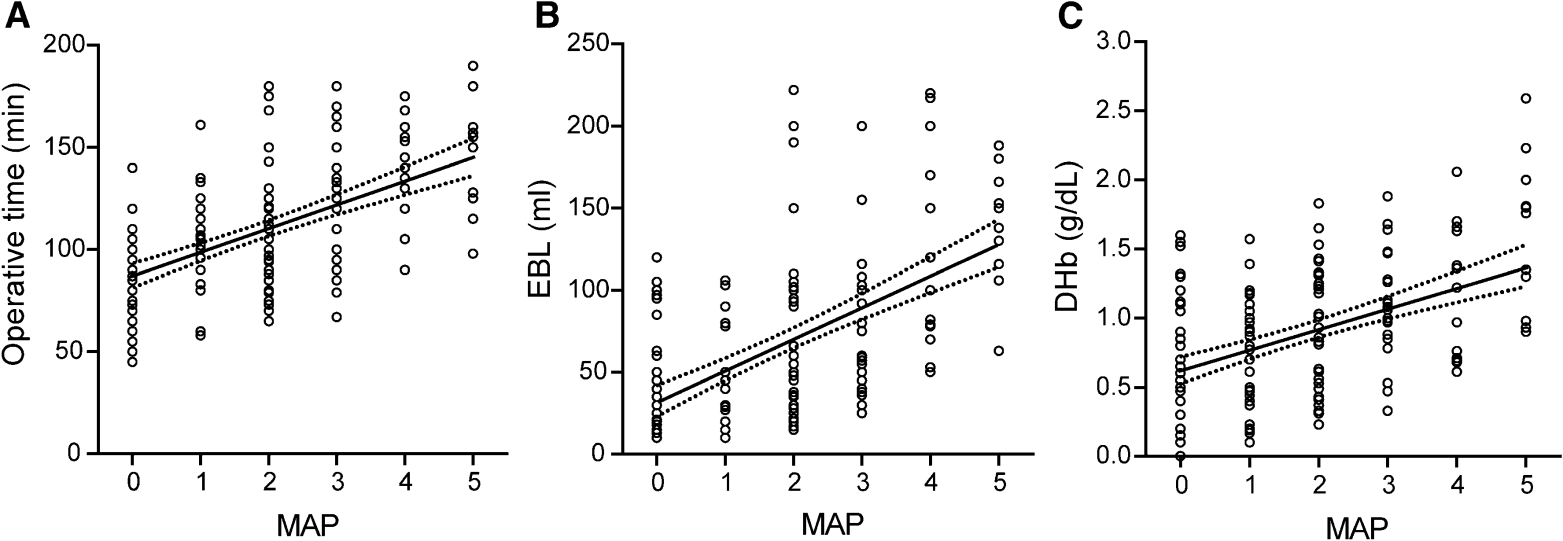
Linear regression between the Mayo scores and perioperative outcomes, including **a** operative time, **b** EBL, and **c** DHb

A total of 186 patients who underwent RLA were included in the analysis. The demographic and clinical characteristics of them are summarized in Table 1. Based on Mayo score, the patients were divided as follows: 0 (*n* = 51, 27.4%), 1 (*n* = 34, 18.3%), 2 (*n* = 45, 24.2%), 3 (*n* = 29, 15.6%), 4 (*n* = 16, 8.6%) and 5 (*n* = 11, 5.9%), respectively. Pathological results are listed. Linear regressions were calculated to predict operative time, EBL and DHb based on Mayo score (Fig. 1).

**Table 1:** Patient, tumor characteristics, and perioperative outcomes

Mean ± SD age (years)	37.3 ± 13.1
*Sex*	
No. male (%)	86 (46.2)
No. female (%)	100 (53.8)
*Laterality*	
No. left (%)	122 (65.6)
No. right (%)	64 (34.4)
Mean ± SD Charlson comorbidity index	1.9 ± 1.7
Mean ± SD BMI (kg/m^2^)	25.0 ± 3.8
Mean ± SD ASA score	1.8 ± 0.8
*Manifestation*	
No. functional (%)	110 (59.1)
No. nonfunctional (%)	76 (40.9)
Mean ± SD operative time (min)	107.6 ± 31.9
Mean ± SD estimated blood loss (mL)	66.6 ± 51.3
Mean ± SD change of hemoglobin (g/dL)^a^	0.9 ± 0.5
Mean ± SD length of drainage (days)	2.1 ± 0.6
*No. Clavien-Dindo complication* (%)	
None	164 (88.2)
Grade I	13 (7.0)
Grade II	8 (4.3)
Grade IVa	1 (0.5)
Mean ± SD tumor diameter (cm)	2.1 ± 1.0
*No. tumor pathology* (%)	
Adenoma	156 (83.9)
Adrenal cyst	3 (1.6)
Nodular hyperplasia	19 (10.2)
Myelolipoma	8 (4.3)
Mean ± SD Mayo score	1.8 ± 1.5
*No. Mayo score* (%)	
0	51 (27.4)
1	34 (18.3)
2	45 (24.2)
3	29 (15.6)
4	16 (8.6)
5	11 (5.9)

^a^The reference range of hemoglobin values: 13–17.5 g/dL (adults)

For easier interpretation of the results, the scores were then artificially stratified into low-risk (0–1), moderate-risk (2–3) and high-risk (4–5) groups. As shown in Table 2, longer operative time (92.0 ± 25.0 vs. 114.7 ± 30.6 vs. 137.4 ± 27.1 min, *P* < 0.001), higher EBL (42.2 ± 28.1 vs. 70.5 ± 44.9 vs. 132.6 ± 63.4 mL, *P* < 0.001), and more DHb (0.7 ± 0.4 vs. 1.0 ± 0.4 vs. 1.3 ± 0.6 g/dL, *P* < 0.001) were significantly associated with increased Mayo score risks. However, no difference was found between the groups with respect to complication rates.

**Table 2:** Perioperative outcomes according to the three risk groups of Mayo score

Outcomes	Mayo score risk			*P*
	Low (0–1, *n* = 85)	Moderate (2–3, *n* = 74)	High (4–5, *n* = 27)	
Operative time, min, mean ± SD	92.0 ± 25.0	114.7 ± 30.6	137.4 ± 27.1	<0.001^a^
EBL, mL, mean ± SD	42.2 ± 28.1	70.5 ± 44.9	132.6 ± 63.4	<0.001^a^
DHb, g/dL, mean ± SD	0.7 ± 0.4	1.0 ± 0.4	1.3 ± 0.6	<0.001^a^
Complications, +/-	9/76	9/65	4/23	0.890^b^

^a^ANOVA

^b^Pearson chi-squared

No major intraoperative complications or open conversion occurred, except in one patient with inferior vena cava injury sutured with 4–0 Prolene. 22 (11.8%) postoperative complications were recorded without reintervention. Low-grade (grade I–II) postoperative complications such as wound infection, subcutaneous emphysema and pneumonia occurred in 21 (11.3%) patients. High-grade (grade IVa) complications occurred in only one patient with a Mayo score of 3 for ventricular fibrillation during extubation and recovered after cardiopulmonary resuscitation and defibrillation. No blood transfusions were required. No residual tumor or relapse was observed during a median follow-up of 24 months.

The association of potential factors with Mayo score was analyzed. Age, male and BMI positively correlated with it (Table 3). Table 4 summarizes the associations of clinical factors with perioperative results. On univariable analysis, age, sex, BMI and Mayo score were associated with operative time (Table 4A), age, sex and Mayo score with EBL (Table 4B), and sex as well as Mayo score with DHb (Table 4C). However, on multivariable analysis, Mayo score was the only factor associated with operative time, EBL and DHb simultaneously. Conversely, only CCI, but not Mayo score, was significantly correlated with complications (Table 4D).

**Table 3:** Association of variables with Mayo score in RLA

Variables	Mayo score (mean ± SD)	Association with Mayo score	
		OR (95% CI^a^)	*P*
*Age, years*			
<50	1.6 ± 1.4	1.00	
≥50	2.7 ± 1.6	1.66 (1.29–2.16)	<0.001
*Sex*			
Female	1.3 ± 1.3	1.00	
Male	2.3 ± 1.5	1.60 (1.29–1.98)	<0.001
*BMI (kg*/*m*^2^)			
<25	1.3 ± 1.4	1.00	
25–30	2.4 ± 1.5	1.69 (1.33- 2.13)	<0.001
>30	2.5 ± 1.4	1.73 (1.22–2.45)	0.002
*Lesion size* (cm)			
<3	1.8 ± 1.5	1.00	
≥3	1.9 ± 1.5	1.02 (0.79–1.26)	0.982
*Manifestation*			
No	1.8 ± 1.3	1.00	
Yes	1.9 ± 1.4	1.19 (0.55–2.59)	0.681
*CCI*			
<2	1.4 ± 1.3	1.00	
2–4	2.1 ± 1.7	1.22 (0.48–3.01)	0.713
>4	2.2 ± 1.5	2.16 (0.57–8.36)	0.255

^a^*CI* confidence interval

**Table 4:** Univariable and multivariable analysis evaluating the correlation of clinical parameters with operative time (A), EBL (B), DHb (C) and complication (D)

	Univariable analyses			Multivariable analyses		
	Coefficient	95% CI^a^	*P*	Coefficient	95% CI	*P*
(A) *Operative time* ^b^						
Age	0.74	0.40 to1.07	< 0.001	0.35	0.04 to 0.66	0.026
Sex	− 20.63	− 29.42 to − 11.84	< 0.001	− 10.19	− 18.21 to − 2.18	0.013
BMI	1.82	0.64–3.00	0.003	0.56	− 0.49 to 1.61	0.295
CCI	3.83	− 0.07 to 7.65	0.061	0.90	− 5.23 to 7.27	0.820
Mayo score	11.60	9.03 to 14.18	< 0.001	9.06	6.12 to 12.00	< 0.001
(B) *EBL*^b^						
Age	1.01	0.46 to 1.56	< 0.001	0.29	− 0.21 to 0.79	0.249
Sex	− 22.24	− 36.82 to − 7.67	0.003	− 3.72	− 16.77 to 9.33	0.574
BMI	1.59	− 0.35 to 3.53	0.108	− 0.754	− 2.46 to 0.95	0.385
CCI	2.58	− 12.10 to 17.62	0.067	6.13	− 5.78 to 16.67	0.377
Mayo score	19.21	15.13 to 23.30	< 0.001	18.57	13.78 to 23.36	< 0.001
(C) *DHb*^b^						
Age	0.03	− 0.03 to 0.08	0.352	–	–	–
Sex	− 1.64	− 3.06 to − 0.23	0.023	− 0.76	− 2.10 to 0.58	0.691
BMI	0.09	− 0.10 to 0.27	0.368	–	–	–
CCI	0.31	− 1.15 to 1.65	0.640	–	–	–
Mayo score	1.52	1.09 to 1.94	< 0.001	1.38	1.02 to 1.75	0.003

	OR	95% CI	*P*	OR	95% CI	*P*
(D) *Complication*^c^						
Age	1.01	0.98 to 1.05	0.501	–	–	–
Sex	0.39	0.15 to 1.06	0.064	0.25	0.06 to 1.73	0.385
BMI	1.10	0.98 to 1.22	0.099	1.41	0.68 to 2.19	0.521
CCI	4.37	1.78 to 11.25	0.001	1.83	1.12 to 4.78	0.014
Mayo score	1.09	0.82 to 1.46	0.550	–	–	–

^a^*CI* confidence interval

^b^Linear regression model

^c^Logistic regression model

## Discussion

RLA has gained increasing popularity with comparable outcomes to transperitoneal approach but less interference with intra-abdominal organs [15]. Although RLA allows direct access to the kidney and adrenal gland, it is a technically demanding procedure because of the relatively small working space, which can be aggravated by redundant perinephric fat [6]. To our knowledge, this is the first report utilizing a scoring system to comprehensively evaluate the status of periadrenal fat and report the relationship between Mayo score and perioperative outcomes of RLA. Our data demonstrated that only Mayo score was an independent risk factor for operative time, EBL and DHb. Here, we introduced the parameter DHb to evaluate perioperative blood loss including possible excessive postoperative bleeding, which would be underestimated by routine EBL. However, we did not find a correlation between Mayo score and complication. A large-scale retrospective analysis of adrenalectomy revealed that intraoperative blood transfusion was an independent predictor of complications, especially in patients with operative time > 150 min [16]. Given that RLA is associated with a low complication rate, operative time could be used as a surrogate for surgical complexity as well as an indicator of complication risk. In our cohort, the increasing operative time and blood loss along with Mayo score did not translate into significant difference of complications. This could be attributed to the limitations of the study including the relatively small tumor size and cohort. Several clinical variables were analyzed to clarify their association with Mayo score. And age, sex and BMI achieved significance, similar to previously reported [14].

The Mayo score has been developed to scale the possibility of adherent perinephric fat, reflecting both the quantity and texture of it [14]. Generally, adrenal gland is encapsulated in perinephric fat with its medial surface adjacent to peritoneum. Based on the anatomical features, obesity and abundant visceral fat were recognized as adverse conditions to perform laparoscopic adrenalectomy [17, 18]. Compared to lean patients, overweight patients sustained increased operative time and greater probability of complications [5]. Recently, it was demonstrated that anthropometric measurements, specifically periadrenal fat volume and operative laterality, were better predictors of increased operative time than BMI in RLA [19]. However, Kazaryan et al. revealed that moderately increased operative time was the only perioperative parameter with significant difference between obese and lean patients [20]. Hu et al. found that RLA offered similar complication rates in patients with different obesity status [8]. The controversy may be explained by the long span of survey, diverse technique maturity and concentration only on fat volume [7, 19, 21]. Hence, further improvement should be made to elucidate the exact role of periadrenal fat in RLA due to the following points. First, the obesity status or BMI alone cannot represent the fat volume surrounding the adrenal gland. Erbil et al. showed that retroperitoneal fat mass was more useful than BMI in predicting adrenalectomy outcomes [22]. Second, not only the quantity but also the texture of fat would affect the procedure, implying the need of concerning both of the factors for the evaluation of periadrenal fat. We noticed that redundant perinephric fat substantially complicated the manipulation, especially dissection of the first and third avascular planes. When adherent fat existed, it was particularly difficult to identify kidney capsule, which can cause damage to the kidney or adrenal gland. The Mayo score was developed as a potential candidate tool for the evaluation. The “out of scope” utilization of this system in our study was based on the following fundamental concepts. Kidney shares the same fat capsule with adrenal gland. Posterior perinephric fat thickness, one parameter of Mayo score measured at the level of the renal vein, is adjacent to the inferior part of the gland. This is parallel to the recently reported quantitative tool of RLA, posterior adiposity index [21]. Furthermore, the existence of fat stranding affects the dissection of the third plane and inferomedial adrenal gland. Therefore, it seems rational to use Mayo score to assess the periadrenal fat status.

For patients with adherent perinephric fat or high Mayo scores, it would be difficult to distinguish the profile of adrenal gland, except for its medial surface, which barely possesses loose areolar tissue that is hardly affected by adherent fat. To achieve satisfactory results, beyond the experience of surgeons, an appropriate surgical plan and dissection route should be considered. Technically, there are two routes for RLA: the en bloc and the split [4, 23]. The en bloc route emphasizing the initial entire dissection of periadrenal fat from the surrounding muscles enables early identification of the medial adrenal surface, avoiding blind dissection in the massive fat and inadvertent injury to the adjacent structures. Furthermore, this strategy facilitates the recognition of upper renal pole in fat tissue, with retraction provided by the adrenal gland. The entire cohort underwent the en bloc route. Notably, another study showed serious saponification of perinephric fat and heavy adhesion to renal fascia led to the transformation of the split route to the en bloc in three cases, suggesting that the en bloc route would be preferred in the setting of adherent fat [24]. Taken together, these findings support the potential value and promising application of Mayo score as an omnidimensional measurement of periadrenal fat for operative planning, preoperative evaluation and consultation.

In our study, to reduce potential bias, patients with maximal tumor diameters > 5 cm, pheochromocytomas or paragangliomas were excluded. The periadrenal fat of pheochromocytomas and paragangliomas has distinct characteristics compared to that of adenoma. Catecholamine secreted by them is an important regulatory factor for lipolysis. The abdominal visceral fat area was reported to be significantly lower in patients with pheochromocytomas than in those with nonfunctioning adenomas [25]. The majority of these patients exhibit phenotypic browning in periadrenal fat accompanied by metabolic alterations [26]. Longer operative time for pheochromocytomas compared to other adrenal tumors has been reported [27]. In addition, it has been well documented that pheochromocytoma and large tumor size were independent risk factors for the perioperative complications [28, 29].

This study has several limitations, including its retrospective nature, relatively small cohort and single-institutional design. Due to the low proportion of patients with high Mayo scores (less than 10% of score 5), the comparisons were likely underpowered. And the predictability of Mayo score for perioperative complications may be underestimated. Larger cohorts, external validation, multi-institution collaborative efforts and prospective studies are needed to confirm our results. In addition, because of the indefinite measuring points and the uneven distribution of perinephric fat, measurement of its posterior thickness has an inherent bias, as well as the evaluation of stranding type due to lack of objective standards, which warrants further studies to improve the metrics.

## Conclusions

The Mayo score, a quantitative system readily measured through preoperative tomography imaging, has been shown to be an independent predictor of perioperative outcomes in RLA. It can be used in the surgical planning for patients undergoing RLA.
